# Unveiling the influence of hip isokinetic strength on lower extremity running kinematics in male national middle-distance runners: a correlational analysis

**DOI:** 10.1186/s13102-024-00946-x

**Published:** 2024-07-19

**Authors:** Ozan Sever, Yunus Öztaşyonar, Halil İbrahim Ceylan, Bülent Okan Miçooğullari, Ryland Morgans, Nicola Luigi Bragazzi

**Affiliations:** 1 Faculty of Sport Sciences, Ataturk University, Erzurum, 25240 Turkey; 2 Physical Education and Sports Teaching Department, Kazim Karabekir Faculty of Education, Ataturk University, Erzurum, 25240 Turkey; 3 Faculty of Sport Sciences, Nevsehir Haci Bektas University, Nevşehir, Turkey; 4 School of Sport and Health Sciences, Cardiff Metropolitan University, Cardiff, UK; 5 Laboratory for Industrial and Applied Mathematics (LIAM), Department of Mathematics and Statistics, York University, Toronto, Canada; 6 Human Nutrition Unit (HNU), Department of Food and Drugs, Medical School, University of Parma, Building C, Via Volturno, 39, Parma, 43125 Italy

**Keywords:** Hip strength, Running kinematics, Knee, Hip, Male, Runners

## Abstract

**Background:**

The relationship between hip strength deficiency in various planes and musculoskeletal injuries within the movement system has been well-established in numerous studies. The present study sought to explore the relationships between hip strength and specific aspects of lower extremity running kinematics.

**Methodology:**

To achieve this objective, the three-dimensional running kinematics of 21 male elite middle-distance runners (mean age: 19.7 ± 1.2 years; mean experience 6.5 ± 1.0 years) were assessed using nine high-speed cameras on a treadmill at a speed of 16 km·h⁻¹. Concurrently, isokinetic hip strength was measured at a speed of 60 deg·s⁻¹ in both the dominant and non-dominant legs. The Pearson correlation coefficient and Paired Samples t-test were utilized.

**Results:**

While no significant differences were found in several isokinetic strength measurements, notable differences in running kinematics were observed. Specifically, pelvic drop at midstance (MS) was significantly lower in the DL (5.79 ± 3.00°) compared to the NDL (8.71 ± 1.39°) with a large effect size (t=-4.04, *p* < 0.001, Cohen’s d = 1.25). Additionally, knee adduction at maximum showed a moderate effect size difference, with the DL at 2.99 ± 1.13° and the NDL at 3.81 ± 1.76° (t=-2.74, *p* = 0.03, Cohen’s d = 0.55). Results indicated a moderate to highly positive association between running knee adduction in the dominant leg and hip external rotation (*r* = 0.67, *p* < 0.05), concentric extension (*r* = 0.77, *p* < 0.05), and concentric abduction (*r* = 0.78, *p* < 0.05). Additionally, the running tibial external rotation angle in the dominant leg exhibited an inverse relationship with all strength measurements, with statistical significance observed only for concentric extension force (*r*=-0.68, *p* < 0.05). Furthermore, hip internal rotation force demonstrated a highly inverse correlation with foot pronation in the dominant leg (*r*=-0.70, *p* < 0.05) and anterior pelvic tilt in the non-dominant leg (*r*=-0.76, *p* < 0.05).

**Conclusions:**

These findings underscore the interrelation between hip strength and running kinematics, particularly on the dominant side. In light of these observations, it is imperative to consider hip strength exercises as integral components for correcting running kinematics. Coaches should also be mindful that kinematic deviations contributing to running injuries may manifest unilaterally or specifically in the dominant leg.

## Background

The proper alignment and coordination of the lumbo-pelvic-hip structure around the pelvis, which is regarded as the core structure, is essential for many locomotor skills, such as running, walking, and jumping [1]. Lumbo-pelvic-hip complex abnormalities in athletes are associated with many athlete injuries. Among these, hip muscle strength is paramount to explaining the lower extremity dysfunctions related to the lumbo-pelvic-hip complex [2–4]. For optimal force production, co-activation of the gluteus maximus and synergistic gluteal muscles provides effective lower extremity movement [5]. Consequently, a substantial body of literature has investigated the link between hip strength and overuse injuries in running [6, 7]. An examination of the existing literature encompassing biomechanical and clinical investigations reveals that compromised muscular coordination within the hip, pelvis, and trunk can exert significant influences on the kinematics and kinetics of the tibiofemoral and patellofemoral joints across various planes of motion [7].

Recent longitudinal cohort studies have significantly advanced our understanding of these risk factors. For instance, the 12-month prospective “Running Injury Surveillance Centre” (RISC) study identified that high training volumes, sudden increases in intensity, and poor running mechanics are significant contributors to running-related injuries (RRIs)​ [8]. Similarly, a study on Dutch runners emphasized the acute workload ratio (ACWR) as a critical predictor of injuries, finding that maintaining an optimal ACWR reduces injury risk, while deviations increase susceptibility to injuries​ [9]. Furthermore, a systematic review and meta-analysis highlighted that biomechanical factors, such as excessive pronation and inadequate hip strength, and musculoskeletal assessments like muscle strength and joint range of motion, are vital in predicting RRIs​ [10]. The TRAIL prospective cohort study focused on runners with a history of knee surgery or early osteoarthritis, suggesting that consistent running load, proper biomechanics, and muscle strength are essential in maintaining knee health and preventing the progression of osteoarthritis symptoms​ [11]​. Additionally, Saragiotto et al. (2014) found that novice runners enrolled in systematic training programs showed predictors of running-related injuries related to training design and biomechanical factors​ [12]​. Van Middelkoop et al. (2008) emphasized risk factors among marathon runners, particularly highlighting the importance of training intensity and pre-existing health conditions​ [13]​. These comprehensive findings from various studies underscore the multifactorial nature of injury risk factors, from training loads to biomechanical integrity, and the importance of longitudinal data in developing effective injury prevention strategies.

The dysfunctions in runners can arise from abnormal sequencing of muscle activation patterns, impaired muscular balance, and lack of mobilization/stabilization. These impairments harm running mechanics [14, 15]. Biomechanical abnormalities in the pelvis, such as excessive anterior pelvic tilt, excessive or restricted obliquity, and asymmetric movements, have been implicated in many running-related injuries [16, 17]. These dysfunctions are generally called lower-crossed syndrome [18]. Also, it can be seen when dynamic movements are performed on one leg with compensatory movements such as excessive anterior pelvic tilt, hip internal rotation and adduction (pelvic drop), an increase in the abduction angle of the knee joint, excessive tibial rotation and excessive pronation [18–21]. In another study, these lower extremity kinetic chain abnormalities have been explained as excessive pronation, knee abduction, femoral internal rotation, and tibial external rotation in functional movements [22].

The aforementioned excessive joint movements manifest predominantly during the absorption phase of the stance cycle in running gait, where eccentric force plays a pivotal role in decelerating the descent. Biomechanically during the running gait cycle, absorption continues until the mid-stance phase, which signifies the point where the forward momentum, generated by the ground reaction force, begins to decelerate and the propulsion phase commences. During stance, pronation continues for the initial 20% of this phase [23], resulting in rearfoot eversion and tibial internal rotation (around the talus). This alignment causes the axes of the transverse tarsal joints to align parallelly, securing the forefoot to the ground [24]. Ground reaction force resulting from heel contact (first contact) is distributed along the closed kinetic chain by knee flexion and hip extension. From distal to proximal, the energy distribution is carried upward along the gastrocnemius, which decelerates dorsiflexion; the quadriceps, which decelerates knee flexion; and the gluteus maximus, gluteus medius, and hamstrings which slow down hip flexion [25].

At the hip joint, extensors, external rotators, and adductors eccentrically slow down this one-leg absorption process. In other words, hip flexion, anterior pelvic tilt, hip internal rotation, and hip adduction moments resulting from ground reaction force are eccentrically slowed down by muscles such as gluteus maximus and gluteus medius. There is evidence that the inadequate strength, endurance, or working pattern of these muscles is associated with excessive frontal and horizontal plane joint movements that occur in the lower extremity joints during the absorption phase. In literature, previous studies demonstrated negative associations between hip extensor-abductor strength and hip adduction-internal rotation [26], knee valgus [27, 28], and foot pronation [29] while performing single-leg activities.

Accordingly, this study aimed to examine the isokinetic strength of the hip muscles in elite runners that would affect the joint movements in the absorption phase of running. In this context, our study examined the absorption phase of running kinematics, specifically analyzing pelvic drop, anterior pelvic tilt, hip adduction, hip internal rotation, knee adduction, tibial external rotation, and foot pronation angles. These variables were considered potential indicators of injury risk during running. The hypothesis of our study suggests that there will be a positive association between hip strength variables and tibial external rotation, as well as knee adduction variables. Conversely, we anticipate a negative association between hip strength and pelvic drop, anterior pelvic tilt, knee adduction, and foot pronation variables. Furthermore, these associations are expected to be more pronounced in the non-dominant leg.

## Materials and methods

### Participants

Twenty-one national middle-distance (800–3000 m) male runners (mean standard deviation: 19.7 ± 1.2 yrs; experience 6.5 ± 1.0 yrs) attended this controlled laboratory study. The study recruited male elite middle-distance runners who had experience competing at elite levels in middle-distance running events. Participants were required to be free from any lower extremity injuries that might affect running biomechanics and willing to undergo assessments for three-dimensional running kinematics and isokinetic hip strength measurement. Exclusion criteria encompassed female participants and male runners outside the specified age range, individuals with a history of lower extremity surgery within the past year, those with acute or chronic lower limb injuries impacting running mechanics, and participants unable to perform treadmill running at the specified speed due to health reasons. Data collection took place at the Ataturk University Kinesiology and Performance Laboratory. The study protocol underwent meticulous scrutiny and received approval from the Ataturk University, Faculty of Sports Sciences Review Board (approval number: 2021-3), and was executed in strict accordance with the ethical guidelines delineated in the Declaration of Helsinki. Before their involvement, all participants provided written informed consent, with minors securing consent from their parents or legal guardians.

The determination of the minimum sample size for this study was conducted utilizing G-power software 3.1.9.7, developed by Düsseldorf University, Germany [30]. A robust power analysis was undertaken, accounting for the study’s design parameters, namely t-tests and a correlation point biserial model. These considerations encompassed a single group, an α error probability set at 0.05, a power of 0.80 (1-β error probability), and an effect size of 0.50 [26, 31]. The resultant analysis delineated that a cohort of at least 21 participants would be indispensable to attain the desired power of 81.72%.

### Procedures

Two main measurement outcomes were obtained for each participant in this study: isokinetic hip peak torque [Nm·kg^− 1^] and pelvic and lower extremity running angular kinematics.

### Isokinetic strength

Before isokinetic tests, the athletes engaged in a 10-minute supervised warm-up bike rotation at a rhythm of approximately 70 revolutions per minute at an intensity of 70% of the estimated (220-age) maximum heart rate on a stationary cycle ergometer (Monark 828E, Monark Exercise AB, Vansbro, Sweden [32, 33]). Heart rate (HR) was followed by using the HR monitor RS400 (Polar Electro, Kempele, Finland). After the bicycle ergometer ride, lumbo-pelvic-hip complex muscles were actively engaged using a previously implemented protocol, lasting approximately 5–7 min of further warm-up. This protocol consisted of exercises such as the double-leg bridge, side-lying hip abduction, quadruped lower extremity lift, gluteal clam with 60° hip flexion, dirty dog (quadruped hip abduction), single-leg hip extension, and stability ball wall squat. The regimen above was detailed in a previous study by Crow et al. [34]. This warm-up routine was implemented to prepare the athletes for subsequent isokinetic tests.

Five minutes following the completion of the warm-up, athletes underwent isokinetic measurements. The isokinetic testing protocol, administered bilaterally at an angular velocity of 60°·s^− 1^, utilized a dynamometer (IsoMed 2000, D&R Ferstl, Hemnau, Germany) and comprised three tests: hip extension (concentric and eccentric), hip abduction (concentric and eccentric) and hip internal and external rotation (concentric). Consequently, the forces of the gluteal muscles, specifically the hip extensors and abductors, were measured in both concentric and eccentric phases. Tests were conducted with alternating dominant and non-dominant leg sequences to mitigate potential fatigue within the same limb. To determine the dominant foot of a participant, the following question, which is reported to be a valid method in a recent systematic review and meta-analysis study [35], was asked: “Which foot do you prefer to kick the ball with?” [36]. Before the isokinetic tests, subjects performed 10 low-intensity dynamic repetitions to familiarise themselves with the motion characteristics. Each test consisted of 2 sets, each comprising 5 repetitions with a 1-minute rest interval between sets. For instance, a participant performing the isokinetic hip extension test on the dominant leg repeated the same test one minute later. Subsequently, the same test was conducted for two sets on the non-dominant leg. This sequencing was employed to prevent fatigue within the same limb.

During the hip extension evaluation, participants assumed a supine position upon the seat of the dynamometer apparatus. A cushioned attachment encircled the thigh of the examined limb, with additional straps employed to stabilize the pelvis, trunk, and shoulders. The greater trochanter served as the pivotal point for rotation on the dynamometer. Participants adopted a posture with crossed arms positioned anteriorly across the chest, while the range of motion for hip articulation was carefully set from 0° of extension to 120° of flexion [37]. Conversely, for the hip abduction assessment, participants were instructed to recline on their side, maintaining neutral alignment of the pelvis and hip. Stabilizing belts were utilized to secure these regions. The leg under scrutiny was fastened with straps in a fully extended position, whereas the contralateral limb was maintained in a slight knee flexion and stabilized on the examination surface. Measurement procedures were conducted within a defined angular range of 0–45° for hip abduction [38]. In the context of the hip rotation examination, participants assumed a supine position, positioning their feet onto the pedal of the dynamometer while ensuring full knee extension and pelvic neutrality. Subsequently, concentric-concentric movements of hip rotation, encompassing medial and lateral rotations, were performed within a designated angular range of approximately 35° for medial rotation and 45° for lateral rotation.

To ensure maximal voluntary contraction during the tests, athletes were verbally encouraged. The peak torque scores within the two sets were normalized to body weight and recorded as hip strength in Nm·kg^− 1^ (as illustrated in Table 1). All tests were implemented by professional operators following operating instructions in the Isomed 2000 dynamometer user manual.

### Running kinematics

During treadmill running, the three-dimensional kinematics of the participants were collected at a rate of 250 Hz utilizing a 9-camera motion analysis system manufactured by Qualisys AB, headquartered in Gothenburg, Sweden. The lower extremity sports marker set from Qualisys (Qualisys AB, Sweden) was used. It consisted of nineteen 17 mm diameter tracking reflective markers and two clusters, with each cluster comprising four markers. Bilateral markers delineated key segments of the foot (forefoot 2nd and 5th metatarsal heads, heel at the back-calcaneus), shank (lateral malleolus, tibial tuberosity), thigh (lateral epicondyle, above patella), and pelvis (anterior superior iliac spines [ASIS] and S1). Furthermore, two clusters were securely affixed to the mid-lateral aspect of the femur to precisely track its motion [39].

**Fig. 1:**
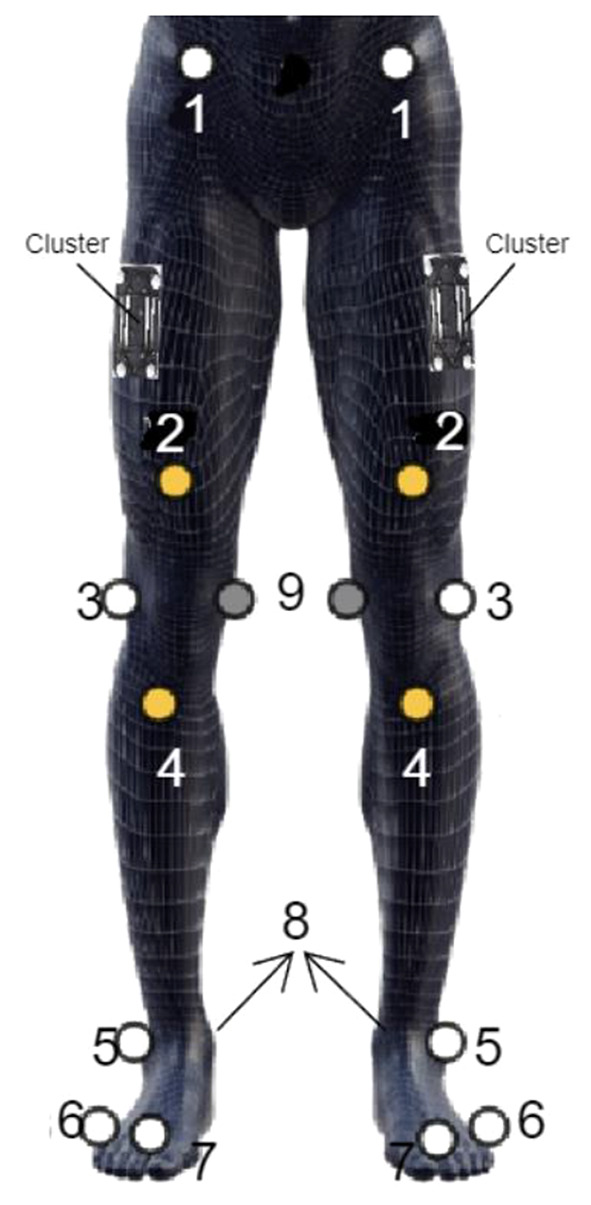
Qualisys Lowers Extremity Sports Marker-Set (Qualisys AB Sweden Qualisys Sports Marker Set [40]. 1, anterior superior iliac spine; 2, one cm proximally of the superior border of the patella when the knee is extended; 3, femur lateral epicondyle; 4, tibial tuberosity; 5, the apex of the lateral malleolus; 6, foot/metatarsus – 5th head; 7, foot/metatarsus – 2nd head; 8, foot/calcaneus – aspect of the Achilles tendon insertion; 9, femur medial epicondyle (static marker/removed while running); 10, a sacrum marker which is not shown in this diagram placed midpoint between left and right posterior superior iliac spine, additionally, two clusters were placed on the lateral half of femur

After static calibration, participants engaged in a 5-minute warm-up jogging and familiarization trials [41]. Subsequently, 3D kinematics were recorded for one minute without participants’ awareness (to prevent potential changes in the running pattern) while running at a speed of 16 km·h^− 1^ on a treadmill (Saturn^®^ 300/100 r, h/p/Cosmos Sports & Medical gmbh, Nussdorf, Germany). The purpose of selecting a relatively low running speed (16 km·h^− 1^) for these athletes was to prolong the weight-bearing duration on the analyzed joints, thereby extending the stance phase and contact time. Trajectories recorded in Qualisys Track Manager software were exported to Visual 3D (C-motion Inc., Kingston, Canada) and Qualisys QTM Project Automation Framework (PAF) Running lower extremity module was used to automatically create a skeletal model, extract running gait cycles, and determine gait events for angular kinematics [39].

The Coda Pelvis model [42] was employed to generate the pelvis segment using markers positioned on ASIS and S1. The hip joint (HIP) center is calculated from the right ASIS, left ASIS, and S1 markers using the predictive equation provided by Bell et al. [43]. The knee joint center was determined using a functional method called SCoRE, employed during the running trial [44]. The SCoRE method uses the markers located on the thigh and the shank. Once the axis of rotation is found, the knee joint center is located at a predicted distance from the lateral femur epicondyle marker using the work of Drillis et al. [45] and Mukhopadhyay et al. [46]. The longitudinal axis of the shank is defined by the knee joint center and the ankle joint center. The ankle joint center is a virtual landmark with an offset (-0.5*(2*Shank_Proximal_Radius * 0.322 + 0.038745) [47] relative to the lateral ankle marker. The orientation of the shank segment is defined by the marker on the tibial tuberosity, which will be anterior relative to the longitudinal axis. The foot segment was delineated by connecting the ankle joint to the marker positioned on the second metatarsal. The marker on the fifth metatarsal was utilized as the lateral reference point to establish the orientation of the foot segment. Kinematic-only foot segments were also created to measure frontal plane ankle kinematics [39].

For each participant, trajectory patterns were filtered at 12 Hz and averaged from the foot strike (0%) to the subsequent foot strike (100%). Foot strike is defined at the local minima of the vertical mid-foot velocity [48]. Midfoot is defined as the midpoint of the calcaneus (8th marker in Fig. 1) marker and the 2nd toe marker (7th marker in Fig. 1). Mid-stance is defined as the first frame when the mid-foot is behind the pelvis (longitudinal axis pointing forward). Toe-off is defined as local maxima in the 2nd toe marker (7th marker in Fig. 1) vertical acceleration [49]. All participants exhibited a running pattern with initial contact on the forefoot and an average ground-foot angle of 17.38° (SD: 3.58, range: 9.4° to 21.8°).Joint kinematics were computed using a Cardan-Euler method with rotations in X (sagittal), Y (frontal), and Z (vertical) planes. Angular values were extracted at mid-stance (MS) during the running stance phase. Stance phase maximal angular values of hip adduction, hip internal rotation (IR), knee adduction, tibial external rotation (ER), ankle pronation, and mid-stance pelvic drop and anterior pelvic tilt were statistically analyzed bilaterally for dominant and non-dominant legs.

### Statistical analysis

The normality of the data about each evaluated variable was assessed through the Shapiro-Wilk test. Paired sample t-tests were used to compare the means between the dominant leg (DL) and non-dominant leg (NDL) for various parameters. Effect sizes were calculated using Cohen’s d, which measures the standardized difference between two means. The interpretation of Cohen’s d effect sizes is classified as follows: values between 0.01 and 0.19 represent a negligible effect, values between 0.2 and 0.49 indicate a small effect, values between 0.5 and 0.79 indicate a moderate effect and values of 0.8 or higher represent a large effect [50]. Subsequently, the Pearson correlation coefficient was utilized to explore the relationships between variables, considering the normality of their distributions. The delineation of correlation magnitudes was explicated as follows: trivial (< 0.10), small (0.10–0.29), moderate (0.30–0.49), large (0.50–0.69), very large (0.70–0.89), nearly perfect (0.90–0.99), and perfect (1), providing a comprehensive framework for interpreting the strength of correlations [51] All statistical analyses were performed using SPSS Version 21 (IBM Corporation, Armonk, USA), with a predefined significance threshold set at *p* ≤ 0.05.

## Results

**Table 1:** The values of hip strength and running kinematic variables (Mean ± S.D.)

	DL	NDL	t	*p*	Cohen’s d
**Isokinetic** **Strength (Nm·kg** ^**− 1**^ **)**					
Hip IR.	0.36 ± 0.09	0.34 ± 0.09	0.84	0.43	0.22
Hip ER.	0.51 ± 0.11	0.49 ± 0.08	1.04	0.37	0.21
Hip Ext. Con.	5.41 ± 0.91	5.84 ± 1.23	-1.5	0.16	0.39
Hip Ext. Ecc.	6.07 ± 1.36	6.22 ± 1.23	-0.8	0.45	0.12
Hip Abd. Con.	1.25 ± 0.36	1.27 ± 0.32	-0.3	0.75	0.06
Hip Abd. Ecc.	1.55 ± 0.43	1.56 ± 0.48	-0.1	0.86	0.02
**Running Kinematics (°)**					
Pelvic Drop (MS)	5.79 ± 3.00	8.71 ± 1.39	**-4.04**	**0.00**	**1.**2**5**.
Pelvic Tilt Anterior (MS)	16.42 ± 4.27	16.02 ± 4.38	0.84	0.42	0.09
Hip Adduction (Max)	11.04 ± 2.96	11.93 ± 2.70	-0.72	0.49	0.31
Hip IR. (Max)	3.84 ± 2.30	5.16 ± 3.50	-0.68	0.52	0.45
Knee Adduction (Max)	2.99 ± 1.13	3.81 ± 1.76	**-2.74**	**0.03**	**0**.**55**
Tibial External Rotation (Max)	1.87 ± 4.97	-1.79 ± 4.02	1.56	0.16	0.81
Ankle Pronation (Max)	9.62 ± 3.12	9.22 ± 4.61	0.50	0.62	0.10

*Notes* IR: internal rotation, ER: external rotation, Con: concentric, Ecc: eccentric, Abd: abduction, MS: mid-stance, Max: maximal angle from first contact to mid-stance, DL: Dominant Leg, NDL: Non-Dominant Leg

Table 1 shows the comparisons of hip strength and running kinematic variables (M ± S.D.) between dominant and non-dominant legs. The isokinetic strength measurements indicated no significant differences between DL and NDL in several parameters. The isokinetic strength measurements indicated no significant differences between the DL and NDL in several parameters. Specifically, Hip IR strength was 0.36 ± 0.09 Nm·kg^− 1^ for DL and 0.34 ± 0.09 Nm·kg^− 1^ for NDL (t = 0.84, *p* = 0.43, Cohen’s d = 0.22, small effect size). Similarly, Hip ER strength was 0.51 ± 0.11 Nm·kg^− 1^ for DL and 0.49 ± 0.08 Nm·kg^− 1^ for NDL (t = 1.04, *p* = 0.37, Cohen’s d = 0.21, small effect size). Hip ext. con. strength showed a slightly higher value for NDL (5.84 ± 1.23 Nm·kg^− 1^) compared to DL (5.41 ± 0.91 Nm·kg^− 1^), though this difference was not significant (t = -1.5, *p* = 0.16, Cohen’s d = 0.39, small effect size). Hip ext. ecc. strength was also similar between DL (6.07 ± 1.36 Nm·kg^− 1^) and NDL (6.22 ± 1.23 Nm·kg^− 1^) (t = -0.8, *p* = 0.45, Cohen’s d = 0.12, small effect size). For hip Abd. strength, concentric and eccentric values were nearly identical between DL and NDL, with concentric strength at 1.25 ± 0.36 Nm·kg^− 1^ (DL) and 1.27 ± 0.32 Nm·kg^− 1^ (NDL) (t = -0.3, *p* = 0.75, Cohen’s d = 0.06, negligible effect size), and eccentric strength at 1.55 ± 0.43 Nm·kg^− 1^ (DL) and 1.56 ± 0.48 Nm·kg^− 1^ (NDL) (t = -0.1, *p* = 0.86, Cohen’s d = 0.02, negligible effect size).

In terms of running kinematics, significant differences were observed in pelvic drop at midstance (MS), with DL showing 5.79 ± 3.00° compared to 8.71 ± 1.39° for NDL (t = -4.04, *p* < 0.001, Cohen’s d = 1.25, large effect size). Pelvic tilt anterior at MS was similar between DL (16.42 ± 4.27°) and NDL (16.02 ± 4.38°) (t = 0.84, *p* = 0.42, Cohen’s d = 0.09, negligible effect size). Hip adduction at the maximum was 11.04 ± 2.96° for DL and 11.93 ± 2.70° for NDL (t = -0.72, *p* = 0.49, Cohen’s d = 0.31, small effect size). Hip IR at maximum was 3.84 ± 2.30° for DL and 5.16 ± 3.50° for NDL (t = -0.68, *p* = 0.52, Cohen’s d = 0.45, small effect size). Knee adduction at maximum showed a significant difference, with 2.99 ± 1.13° for DL and 3.81 ± 1.76° for NDL (t = -2.74, *p* = 0.03, Cohen’s d = 0.55, moderate effect size). Tibial external rotation at maximum was higher for DL (1.87 ± 4.97°) compared to NDL (-1.79 ± 4.02°), although this was not significant (t = 1.56, *p* = 0.16, Cohen’s d = 0.81, large effect size). Lastly, ankle pronation at maximum showed no significant difference between DL (9.62 ± 3.12°) and NDL (9.22 ± 4.61°) (t = 0.50, *p* = 0.62, Cohen’s d = 0.10, negligible effect size).

**Fig. 2:**
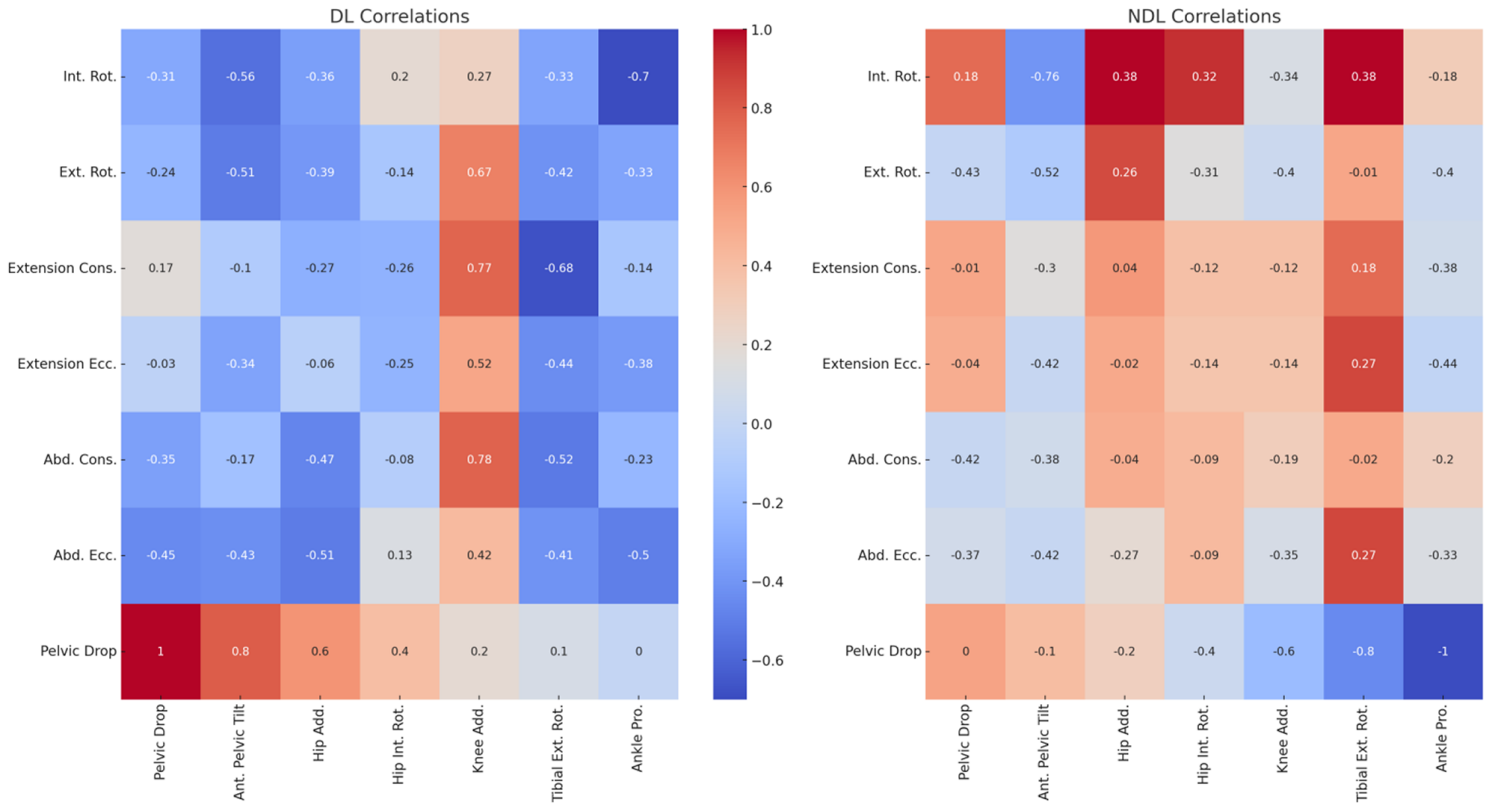
Association between isokinetic hip strength and running kinematics. *Notes* *p < 0.05, Ant: Anterior, Add: Adduction, Int: Internal, Rot: Rotation, Pro: Pronation, Ext: External, Cons: Concentric, Ecc: eccentric, Abd: Abduction, DL: Dominant Leg, NDL: Non-Dominant Leg

In Fig. 2, the heatmaps showing the associations between hip strength and running kinematic variables for both the DL and NDL revealed several notable correlations. For the DL, a significant positive correlation was observed between knee adduction and both hip external rotation (*r* = 0.67, *p* < 0.05, large effect) and hip extension concentric strength (*r* = 0.77, *p* < 0.05, large effect). Additionally, knee adduction was positively correlated with hip abduction concentric strength (*r* = 0.78, *p* < 0.05, large effect). Conversely, tibial external rotation showed a significant negative correlation with hip extension concentric strength (*r* = -0.68, *p* < 0.05, large effect). Ankle pronation was significantly negatively correlated with hip internal rotation strength (*r* = -0.70, *p* < 0.05, large effect). For the NDL, a significant negative correlation was found between anterior pelvic tilt and hip internal rotation strength (*r* = -0.76, *p* < 0.05, large effect). There were no other significant correlations for the NDL, though various parameters showed trends.

## Discussion

The investigation scrutinized synchronized lower-limb kinematics observed throughout the stance phase of running, encompassing anterior pelvic tilt, hip adduction, hip internal rotation, knee abduction, tibial external rotation relative to the femur, and ankle pronation. Through an analysis of the correlation between hip strength and running kinematics, the study aimed to provide significant insights into the biomechanical factors that may influence injury patterns within this athletic cohort.

The main findings of the present study dispelled the initially proposed hypothesis, revealing more significant associations in the dominant leg which suggested lower hip strength in the non-dominant leg. Previous research has highlighted the significance of bilateral force asymmetry in both men and women [52]. However, our investigation demonstrated that the isokinetic strength values of the dominant and non-dominant hips are closely aligned (see Table 1). In our study, it is noteworthy that, although beyond the scope of the current study, statistical analyses did not reveal significant isokinetic hip strength asymmetry between the two legs (Table 1). Intriguingly, there is scant prior research specifically exploring the association between strength and running osteo-kinematics concerning the dominant and non-dominant legs. However, studies on limb asymmetry indicate that kinematic and kinetic asymmetries are common among runners. For instance, in a study involving 63 cross-country runners, it was found that 39 participants exhibited pelvic drop asymmetry between limbs (61.9%); 29 participants showed hip flexion asymmetry (46.0%); 46 participants had hip adduction asymmetry (73.0%); 27 participants displayed knee flexion asymmetry (42.9%); and 31 participants demonstrated ankle dorsiflexion asymmetry (49.2%) [53]. Furthermore, in the same study, the average differences in muscle force impulse between limbs ranged from 27.9 to 114 N.s, with considerable variation in the amount of asymmetry present for each runner. Specifically, 38 participants exhibited asymmetry in gluteus medius impulse (60.3%); 46 participants in gluteus maximus impulse (73.0%); 39 participants in hamstring impulse (61.9%); 28 participants in quadriceps impulse (44.4%); 37 participants in gastrocnemius impulse (58.7%); and 30 participants in soleus impulse (47.6%). Additionally, 27% of the runners demonstrated first-contact ground reaction force asymmetry [53]. Another study examined bilateral asymmetry in runners concerning variables such as running speed and vertical first contact load. In a study with 10 elite, 9 recreational, and 11 novice runners, the overall symmetry index ranged between 0.8% for stride time and 21.4% for vertical average loading rate, regardless of the dominant foot. It was found that among elite athletes, increasing the speed from 8 km/h to 12 km/h reduced the asymmetry in vertical first contact load, indicating that running asymmetry is influenced by running speed [54]. A recent study revealed that running asymmetry was approximately 10% lower at preferred running speeds. The relatively low speed (16 km/h) used in the current study’s procedure might account for the bilateral kinematic asymmetry observed in the athletes [55]. The asymmetry observed in running kinematics but not in isokinetic measurements could be due to the different force production mechanisms arising from the co-activation of multi-joint hip muscles during running, which differs from the isokinetic measurement conditions (conducted in supine position) and speed of isokinetic measurement [56]. This study added a novel dimension to the current body of literature, emphasizing the need for further exploration and understanding of the intricacies of strength and kinematic relationships in running, particularly considering the nuanced dynamics between dominant and non-dominant limbs.

In the dominant leg, our analysis revealed a positive association between hip strength and knee adduction, whereas tibial external rotation and foot pronation exhibited a negative association with hip strength. The knee adduction angle, defining the intersection angle between the longitudinal line through the tibia and the line extending from the knee to the acetabulum, is crucial in understanding lower-limb biomechanics [57]. A negative direction of this angle signifies knee valgus, a position where the knee deviates inwardly. In our study, all athletes maintained positive knee adduction angles in both legs throughout the stance phase of running. The minimum average adduction value during the stance phase was 2.9° in the dominant leg and 3.8° in the non-dominant leg. These minimum adduction values were consistently reached during the mid-stance phase of running. The positive association with knee adduction suggests that greater hip strength correlates with a more favorable knee alignment, potentially mitigating the risk of knee valgus [7, 14, 28, 29, 58]. Conversely, the present study indicated that the negative associations with tibial external rotation and foot pronation highlight the complex biomechanical adjustments in response to hip strength variations during the stance phase of running. These findings contribute valuable insights into the dynamic nature of lower-limb kinematics and their modulation by hip strength, particularly in the context of the dominant leg during running.

The analysis also revealed a strong positive association between knee adduction and all strength measurements, except for hip internal rotation, eccentric abduction, and extension forces. In essence, greater hip strength corresponded to a reduced knee abduction, indicative of diminished dynamic valgus during our experiment. This aligns with existing literature, as prospective [59] and retrospective studies [60] have established links between hip muscle weakness and knee injuries. A study involving cross-country runners exhibited analogous findings for knee frontal plane mobility, where peak isokinetic hip abductor torque inversely correlated with frontal plane hip joint range of motion (ROM) (*r*=-0.462), but not with sagittal or horizontal plane hip ROM [26]. Similarly, peak isokinetic hip extensor torque was associated with hip rotation, not extension and adduction [26]. Hip external rotators (gluteus maximus, deep internal rotators), hip extensors (gluteus maximus, hamstrings, adductor magnus posterior), and hip abductors (gluteus medius, tensor fascia lata, gluteus maximus) have been linked to dynamic knee adduction angle in healthy and athletes population [7, 14, 28, 29, 58, 61].

While the initial hypothesis expected similar associations in hip adduction and internal rotation, the study primarily unveiled significant associations in the knee, tibia, and ankle joints. Notably, in previous research on runners, hip abduction strength was moderately and inversely associated with hip adduction and pelvic internal rotation excursion [16]. In a parallel investigation wherein the inter-play between hip adduction and frontal plane kinematics of the hip during running was examined, a moderate association was identified between hip strength and the relationship of the femoral shaft and femoral neck [6]. In contrast, no discernible association was observed between abductor strength and the adduction angle during the stance phase [6]. In contrast to some previous studies, the present investigation did not find significant associations between hip kinematics and running. This discrepancy may be attributed to insufficient load during the running task, compensatory upper body movements, or potentially differing mechanisms in the specific population studied. For instance, a study implementing a hip extensor and external rotator strengthening program did not alter the running hip kinematics [62]. Still, it did impact pelvic drop, hip adduction, and internal rotation in a single-leg squat exercise. Additionally, the same researchers found no relationship between hip abductor strength and running adduction kinematics. Compensatory mechanisms were observed, including trunk lean to reduce the load on the abductors [62]. Therefore, our study contributes nuanced insights into the intricate relationship between hip strength and lower extremity kinematics during running, emphasizing unique associations in the knee, tibia, and ankle joints, potentially influenced by the specific demands of the running task and individual compensatory mechanisms.

Whereas tibial external rotation angle and foot pronation exhibited an inverse association with all hip strength variables, significant associations were found with concentric extension hip strength for tibial external rotation and hip internal rotation for foot pronation, specifically in the dominant leg (Table 1). This finding implies that lower strength in the gluteus maximus may contribute to tibial external rotation and foot pronation during dynamic movements like running. Although there is limited existing literature investigating the direct relationship between hip strength and tibial kinematics, it is suggested that effective and sufficient hip strength plays a role in providing proximal control over subtalar joint movements [63]. Additionally, the available literature suggests that imbalances in hip muscle strength, particularly lower strength in abductors compared with adductors, are associated with increased foot pronation [64]. Meanwhile, weakness in hip stabilizers, encompassing hip extensors, abductors, and external rotators, may lead to excessive internal hip rotation, inducing foot pronation [65]. Dysfunction in the abductors and external rotators of the hip joint is proposed to result in biomechanical positions associated with foot pronation [66, 67]. Therefore, the study’s findings contribute to our understanding of how hip strength may influence not only hip kinematics but also the effects on tibial rotation and foot pronation during running activities.

Finally, the study revealed an intriguing inverse correlation between pelvic anterior tilt angle and hip internal rotation strength specifically in the non-dominant leg. This finding implies that individuals with lower hip internal rotation strength may exhibit a higher degree of pelvic anterior tilt. The situation can hypothetically be linked to the tightening of the hip’s internal rotator muscles, which can result in an anterior pelvic tilt. Muscles such as the tensor fasciae lata, rectus femoris, and anterior hip adductors may tighten, leading to a loss of strength due to the muscle contracture [67]. The literature does not offer any explanations for this relationship.

This study is subject to several noteworthy limitations that warrant consideration when interpreting its results. A primary limitation is the small sample size, which is primarily attributed to the restricted availability of national long-distance athletes within the country where the research was conducted. The diminutive sample size has the potential to undermine the statistical power of the study, resulting in broader confidence intervals despite the presence of moderate to large correlation coefficients. Another limitation is using a movement speed of 60°·s^-1^ for dynamic isokinetic strength tests. Whereas this speed was chosen to elicit maximal torque, it is essential to acknowledge that different outcomes might be observed at faster movement velocities. Additionally, the study employed a treadmill for running assessments instead of over-ground running. The chosen running speed of 16 km·h^-1^, although within the range of running speeds, is slower than the typical long-distance race pace, potentially impacting the generalizability of the findings. The rationale behind selecting this particular speed was to discern potential compensatory mechanisms by deliberately eliciting a longer stance phase within the running gait cycle. Variables such as ground terrain, shoe type, and shoe selection are recognized factors that may influence running kinematics. It is important to acknowledge that controlling for these variables might yield differing results. Future research with a larger and more diverse participant population, considering various running conditions and variables, is warranted to enhance the generalizability and robustness of the statistical outcomes.

## Conclusion

The results of the present study suggest a partial connection between hip strength and running kinematics, with a particular emphasis on the dominant leg. This highlights the importance of exercises designed to enhance hip strength, as they appear crucial in influencing running kinematics, especially those impacting the knee, tibia, and foot in both the frontal and horizontal planes. Coaches and practitioners should recognize that deficiencies in hip strength may contribute to asymmetrical running biomechanics, potentially leading to greater injury risk. Tailoring interventions to address specific weaknesses in the dominant leg and focusing on exercises that target hip strength may be beneficial in optimizing running mechanics and reducing the likelihood of injuries. Ultimately, these insights contribute to a better understanding of the complex relationship between hip strength and running mechanics. This provides valuable information for coaches, athletes, and practitioners involved in preventing injuries and optimizing running performance.

## Data Availability

The data that support the findings of this study are available on request from the corresponding author.
